# Graspot: a graph attention network for spatial transcriptomics data integration with optimal transport

**DOI:** 10.1093/bioinformatics/btae394

**Published:** 2024-09-04

**Authors:** Zizhan Gao, Kai Cao, Lin Wan

**Affiliations:** Academy of Mathematics and Systems Science, Chinese Academy of Sciences, Beijing 100190, China; School of Mathematical Sciences, University of Chinese Academy of Sciences, Beijing 100049, China; Eric and Wendy Schmidt Center, Broad Institute of MIT and Harvard, Cambridge, MA 02142, United States; Academy of Mathematics and Systems Science, Chinese Academy of Sciences, Beijing 100190, China; School of Mathematical Sciences, University of Chinese Academy of Sciences, Beijing 100049, China

## Abstract

**Summary:**

Spatial transcriptomics (ST) technologies enable the measurement of mRNA expression while simultaneously capturing spot locations. By integrating ST data, the 3D structure of a tissue can be reconstructed, yielding a comprehensive understanding of the tissue’s intricacies. Nevertheless, a computational challenge persists: how to remove batch effects while preserving genuine biological structure variations across ST data. To address this, we introduce Graspot, a **gr**aph **a**ttention network designed for **sp**atial transcriptomics data integration with unbalanced **o**ptimal **t**ransport. Graspot adeptly harnesses both gene expression and spatial information to align common structures across multiple ST datasets. It embeds multiple ST datasets into a unified latent space, facilitating the partial alignment of spots from different slices. Demonstrating superior performance compared to existing methods on four real ST datasets, Graspot excels in ST data integration, including tasks that require partial alignment. In particular, Graspot efficiently integrates multiple ST slices and guides coordinate alignment. In addition, Graspot accurately aligns the spatio-temporal transcriptomics data to reconstruct human heart developmental processes.

**Availability and implementation:**

Graspot software is available at https://github.com/zhan009/Graspot.

## 1 Introduction

Recently, spatial transcriptomics (ST) technologies have enabled the simultaneous measurement of mRNA expression while retaining spatial information within a tissue slice. The integration of multiple slices provides a comprehensive understanding of complex biological processes in entire tissues, fostering innovative approaches to downstream tasks such as analyzing spatial expression across slices and exploring 3D cell–cell communications (Ståhl *et al.* 2016, Baccin *et al.* 2020, Ji *et al.* 2020).

Unlike single-cell batch effect correction, there is no correspondence between spots from different ST slices. Therefore, how to identify and preserve unique biological structural variations during integration remains challenging.

Several methods have been used for alignment and integration of multiple ST slices. For example, PASTE (Zeira *et al.* 2022), which is based on fused Gromov–Wasserstein optimal transport, can align slices in a global manner. However, PASTE assumes that slices have global similarity and fails to consider the biological structure variations across slices. To solve the problem, PASTE2 (Liu *et al.* 2023) extends the single-cell data integration method Pamona (Cao *et al.* 2022b) and introduces a partial fused Gromov–Wasserstein method to select and align only a subset of spots. Meanwhile, both PASTE and PASTE2 work on original space or linear embedding, and cannot provide a common integrated space across ST slices. Moreover, nonlinear embedding based methods are developed, such as GPSA (Jones *et al.* 2023) and STAligner (Zhou *et al.* 2023). GPSA (Jones *et al.* 2023) integrates multiple ST slices into a common coordinate system using deep Gaussian processes, while STAligner (Zhou *et al.* 2023) embeds gene expression and spatial neighbor network of spots with a graph attention network (GAT) and aligns slices using the mutual nearest neighbor (MNN) originally developed for single-cell data integration. However, none of them are capable of handling the partial alignment of slices or providing the probabilistic mapping of spots in adjacent slides for downstream analysis, as achieved by PASTE and PASTE2. There are also some generic methods, such as Tangram (Biancalani *et al.* 2021) and uniPort (Cao *et al.* 2022a), are designed to integrate and align single-cell and ST data. Tangram (Biancalani *et al.* 2021) is a deep learning method that aligns single-cell data onto ST data, while uniPort (Cao *et al.* 2022a) is a unified single-cell data integration framework that combines a coupled variational autoencoder and mini-batch unbalanced optimal transport (UOT). The UOT module allows uniPort more suitable for heterogeneous data integration. However, both Tangram and uniPort fail to utilize the spatial information provided by ST data.

Several existing methods, including STAligner, stack ST slices in a coordinate framework but are limited by requiring the guidance of manual landmarks.

Here we present Graspot, a GAT with UOT that aligns and integrates multiple ST data. Graspot extends uniPort by leveraging both GAT and UOT with the aim to efficiently utilize both gene expression and spatial information provided by ST data for the integration. Graspot inherits the GAT architecture of STAGATE (Dong and Zhang 2022) which nonlinearly embeds both gene expression and spatial information of each slice into a common latent space. The UOT module of Graspot enables it to reconstruct the probabilistic mapping of spots in the latent space across slices (Peyré and Cuturi 2019). Based on the partial probabilistic mapping, Graspot not only efficiently removes the batch effect, but also retains the biological structure variations across slices. We iteratively optimize the GAT and UOT distance to generate both the integrated embedding and probabilistic alignment. Probabilistic alignment result of Graspot can guide the alignment of slices with rotation and translation through weighted iterative closest point (ICP) algorithm in a 3D coordinate framework.

We verified Graspot on human dorsolateral prefrontal cortex (DLPFC) ST data, anterior sagittal mouse brain ST data, HER2 breast cancer ST data, and ST datas of human heart development. Our results indicate that Graspot attains highest alignment accuracy compared to competing methods both on global and partial alignment. Graspot can also integrates multiple ST slices via the pretraining of model. In addition, Graspot automatically align ST slices in a 3D coordinate framework. For instance, Graspot accurately aligns spatio-temporal ST slices to reveal the human heart developmental processes.

## 2 Materials and methods

### 2.1 Overview of Graspot

**Figure 1.**
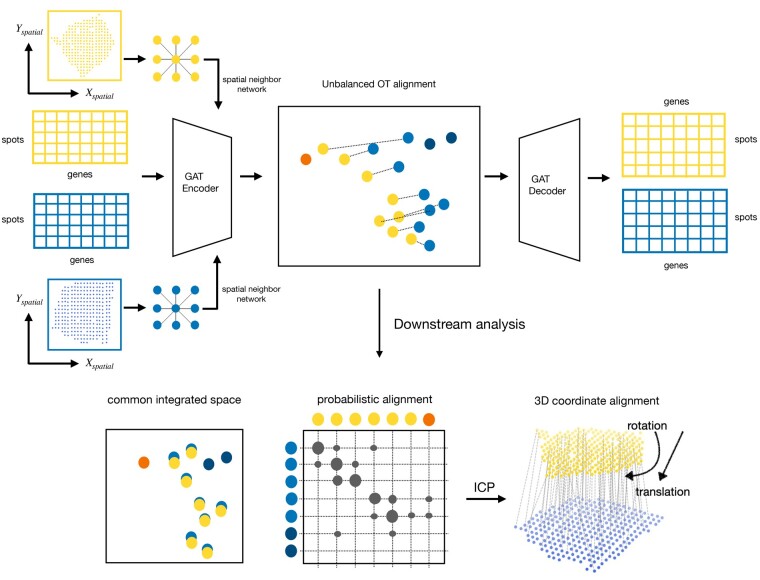
Overview of Graspot algorithm. The inputs of Graspot are multiple spatial transcriptomics slices with gene expression matrices and spatial coordinates. The outputs of Graspot are probabilistic alignment *T* and common integrated space aligns intrinsic structures of multiple ST slices. Probabilistic alignment matrix *T* guides the coordinate alignment using weighted ICP algorithm.

Graspot is a GAT for ST data integration with UOT (Fig. 1). The main inputs of Graspot are multiple ST slices which contain both gene expression profiles and two-dimensional spot coordinates. There are three main outputs of Graspot: (i) a probabilistic alignment of spots across multiple ST slices; (ii) a common low-dimensional space that recovers and aligns multiple ST slices; (iii) 3D coordinate alignment.

### 2.2 Mathematical formulation of Graspot

Suppose that two ST slices $D_{1}=\{(x_{i},s_{i}),i=1,2,\ldots ,n_{x}\}$ and $D_{2}=\{(y_{j},w_{j}),j=1,2,\ldots ,n_{y}\}$ are inputs of Graspot, where $x_{i}\in R^{g}$ is the normalized gene expression of spot *i*, $y_{j}\in R^{g}$ is the normalized gene expression of spot *j* and $s_{i}(w_{j})\in R^{2}$ is the coordinate of spot *i*(*j*). *g* represents for the number of highly variable common genes. Graspot first builds an undirected spatial neighbor network and computes an adjacency matrix **A** based on spot locations in respective slices. Here, $A_{ij}=1$ if and only if the Euclidean distance between spot *i* and spot *j* in same slice is less than a predefined radius *r*. Afterwards, Graspot inputs two ST slices through GAT framework and learns its *k*-dimensional embeddings. The latent vector **z** generated by the graph attention auto-encoder which achieves nonlinear dimensionality reduction while collectively aggregating information from neighbors of spots.

The neural network module in Graspot consists of three parts: an encoder, a decoder, and the graph attention layers. The inputs of encoder are initialing as $h^{\left(0\right)}={\left(x_{1},\ldots ,x_{n_{x}},y_{1},\ldots ,y_{n_{y}}\right)}^{T}$. The encoder in Graspot generates the embedding of spot *i* in *k*th layer as follows:


(1) $$h_{i}^{\left(k\right)}=\sigma \left(\sum_{j\in S_{i}}\left({\text{att}}_{ij}^{\left(k\right)}(W_{k}h_{j}^{\left(k-1\right)})\right)\right),$$

where *σ* is the nonlinear activation function, *S_i_* is the neighbor set of spot *i* according to spatial neighbor network, and $W_{k}$ is weight matrix for training. Besides, Graspot employs a self-attention mechanism, which is an added single-layer neural network in *k*th layer in the encoder, to adaptively learn information from neighbors. The edge weight between spot *i* and spot *j* from same slice is described as follows:


(2) $$e_{ij}^{\left(k\right)}=\sigma \left({\left(v_{s}^{\left(k\right)}\right)}^{T}(W_{k}h_{i}^{\left(k-1\right)})+{\left(v_{r}^{\left(k\right)}\right)}^{T}(W_{k}h_{j}^{\left(k-1\right)})\right),$$

where $v_{s}^{\left(k\right)}$ and $v_{r}^{\left(k\right)}$ are trainable weight vectors, $h_{j}^{\left(k-1\right)}$ is the represent of spot *j* in *k−*1 layer, and att_*ij*_ is normalized form of *e_ij_* via a softmax function as follows:


(3) $${\text{att}}_{ij}^{\left(k\right)}=\frac{\;\text{exp}(e_{ij}^{\left(k\right)})}{\sum_{l\in S_{i}}e\text{xp}(e_{il}^{\left(k\right)})}.$$

After obtaining the latent representations from the encoder, Graspot applies a decoder to reverse the final spot embedding back to the original normalized gene expression. The last layer of the decoder is formulated as follows:


(4) $${\hat{h}}_{i}^{\left(0\right)}=\sigma ({\hat{W}}_{1}{\hat{h}}_{i}^{\left(1\right)}).$$

It has the same number of layers as encoder and we set ${\hat{W}}_{k}=W_{k}^{T}$ and ${\hat{\text{att}}}^{\left(k\right)}={\left({\text{att}}^{\left(k\right)}\right)}^{T}$, respectively, to avoid overfitting. One of the objectives minimized by Graspot is the reconstruction loss of normalized expressions as follows:


(5) $$L_{\text{recon}}=\sum_{i=1}^{N}{||h_{i}-{\hat{h}}_{i}^{\left(0\right)}||}_{2}.$$

To better integrate heterogeneous ST slices in a common low-dimensional space, we further design an alignment term for different ST slices using UOT. Suppose we have two latent vectors $z_{x_{i}}\in {\mathbb{R}}^{k}$ and $z_{y_{j}}\in {\mathbb{R}}^{k}$ generated by encoder *ψ*, respectively. Due to the favorable properties of the common latent space that recover intrinsic structures of ST slice, the UOT cost between spot *i* and spot *j* in different slices is defined as Euclidean distance of latent vectors $z_{x_{i}}$ and $z_{y_{j}}$ as follows:


(6) $$C_{ij}=||z_{x_{i}}-z_{y_{j}}{\left|\right|}^{2}.$$

We then compute the following unbalanced entropic optimal transport plan (Benamou 2003):


(7) $$\begin{array}{c} T^{*}=\arg\underset{T\in R_{+}^{n_{x}\times n_{y}}}{\min}\langle C,T\rangle -\epsilon H(T)+\\ \rho_{1}D_{KL}(T1_{n_{y}}||a)+\rho_{2}D_{KL}(T^{T}1_{n_{x}}||b) \end{array},$$

where $\langle C,T\rangle =\sum_{i,j}C_{ij}T_{ij}$, entropy regularization term $H(T)=-\sum_{i,j}T_{ij}(\text{log}\;T_{ij}-1)$, and $a=\frac{1}{n_{x}}1_{n_{x}},\;b=\frac{1}{n_{y}}1_{n_{y}}$. *ϵ* is an entropy parameter. *ρ*_1_ and *ρ*_2_ are two regularization parameters. As mentioned above, multiple ST slices may only partially overlap, and as a result, there may be a portion of spots in both slices that are seldom aligned. It is worth noting that the UOT module in Graspot allows only a fraction of the total mass to be transported between two distributions. Equation (7) combines entropic regularization (Cuturi 2013) with a more general formulation for UOT. Unbalanced entropic optimal transport allows to slightly diverge from initial mass during transportation and can be optimized using variations of the Sinkhorn algorithm (Janati *et al.* 2020). This is a strictly convex optimization problem and can be solved via an efficient inexact proximal point method (Xie *et al.* 2020) as:


(8) $$\alpha^{\left(l+1\right)}={\left(\frac{a}{G\beta^{\left(l\right)}}\right)}^{\frac{\rho_{1}}{\rho_{1}+\epsilon }},$$

(9) $$\beta^{\left(l+1\right)}={\left(\frac{b}{G^{T}\alpha^{\left(l\right)}}\right)}^{\frac{\rho_{2}}{\rho_{2}+\epsilon }}.$$

The initial $\beta^{\left(0\right)}=\frac{1}{n_{x}}1_{n_{x}},\;T_{ij}^{\left(0\right)}=\frac{1}{n_{x}n_{y}}1_{n_{x}n_{y}}$ and $G_{ij}=T_{ij}^{\left(l\right)}\;\text{exp}\;\left(-\frac{C_{ij}}{\epsilon }\right)$, and the final UOT plan is calculated as $T_{ij}^{*}=\alpha_{i}^{*}G_{ij}^{*}\beta_{j}^{*}$. The second objective of our method is defined as the UOT loss:


(10) $$L_{\text{UOT}}=\sum_{i,j}C_{ij}\times T_{ij}^{*}.$$

Therefore, the total objective minimized by Graspot is formulated as:


(11) $$L_{\text{total}}=(1-\gamma )L_{\text{Recon}}+\gamma L_{\text{UOT}}.$$

Here, *γ* is the trade-off of reconstruction loss and UOT loss. We set γ=0.2 in the following experiments.

We perform UOT and graph attention auto-encoder training in an iterative manner to generate a probabilistic optimal transport plan and a common aligned low-dimensional space. Graspot provides users an option to initialize $T^{\left(0\right)}$ in the optimization of Equation (7) to enhance the participation of spatial information in alignment module. Finally, the optimal transport plan **T** produced as the output represents the probabilistic correspondence between spots. The encoder’s output is a commonly aligned low-dimensional space for multiple ST slices. Details of Graspot algorithm are shown in Supplementary Note S1.

Once probabilistic alignment **T** is obtained, the source slice would be transformed to the target slice according to the optimal rotation and translation solution from SVD-based weighted ICP optimization in coordinate framework (Supplementary Note S3).

## 3 Methods evaluation

We evaluate our method both from the spots mapping **T** for alignment and nonlinear integrated embedding **z**. We mainly employ the metrics of Alignment accuracy and Label Transfer ARI to assess the performance of spots mapping **T** for alignment. Given spots mapping $T_{ij}$, we compute the maximum probability matching $M_{ij}$ and one-to-one spot correspondence. Alignment accuracy measures the fraction of matched spots belonging to the same cell types across slices. Label Transfer Adjusted Rand Index (LTARI) indicates the overlap of annotated cell types and corresponding cell types. To evaluate nonlinear integrated embedding **z**, we employ the metrics of two categories including removal of batch effects and conservation of biological structure variations. Here the former category, including Batch Entropy score and Batch Average Silhouette Width (Batch ASW), refers to measure batch mixing. The latter category, including Silhouette (Cell-type ASW) score, is used to determine the separation of cell types. Both metrics work on the basis of the common embedded space of the integrated datasets (See Supplementary Note S2).

## 4 Results

### 4.1 Graspot outperforms the state-of-the-art methods on global alignment with highest accuracies

We applied Graspot to analyze 10× Genomics Visium ST data from human DLPFC. This dataset consists of 12 slices of DLPFC tissue from three samples, denoted as I, II, and III. Each sample consists of four slices labeled from A to D along the *z*-axis. Within each sample, the first adjacent pair AB and the last adjacent pair CD are 10 µm apart, whereas the middle pair BC of slices is located 300 µm apart (Maynard *et al.* 2021). The slices were manually annotated into six neocortical layers plus a white matter by Maynard *et al.* (2021). We used the annotation for method evaluations.

**Figure 2.**
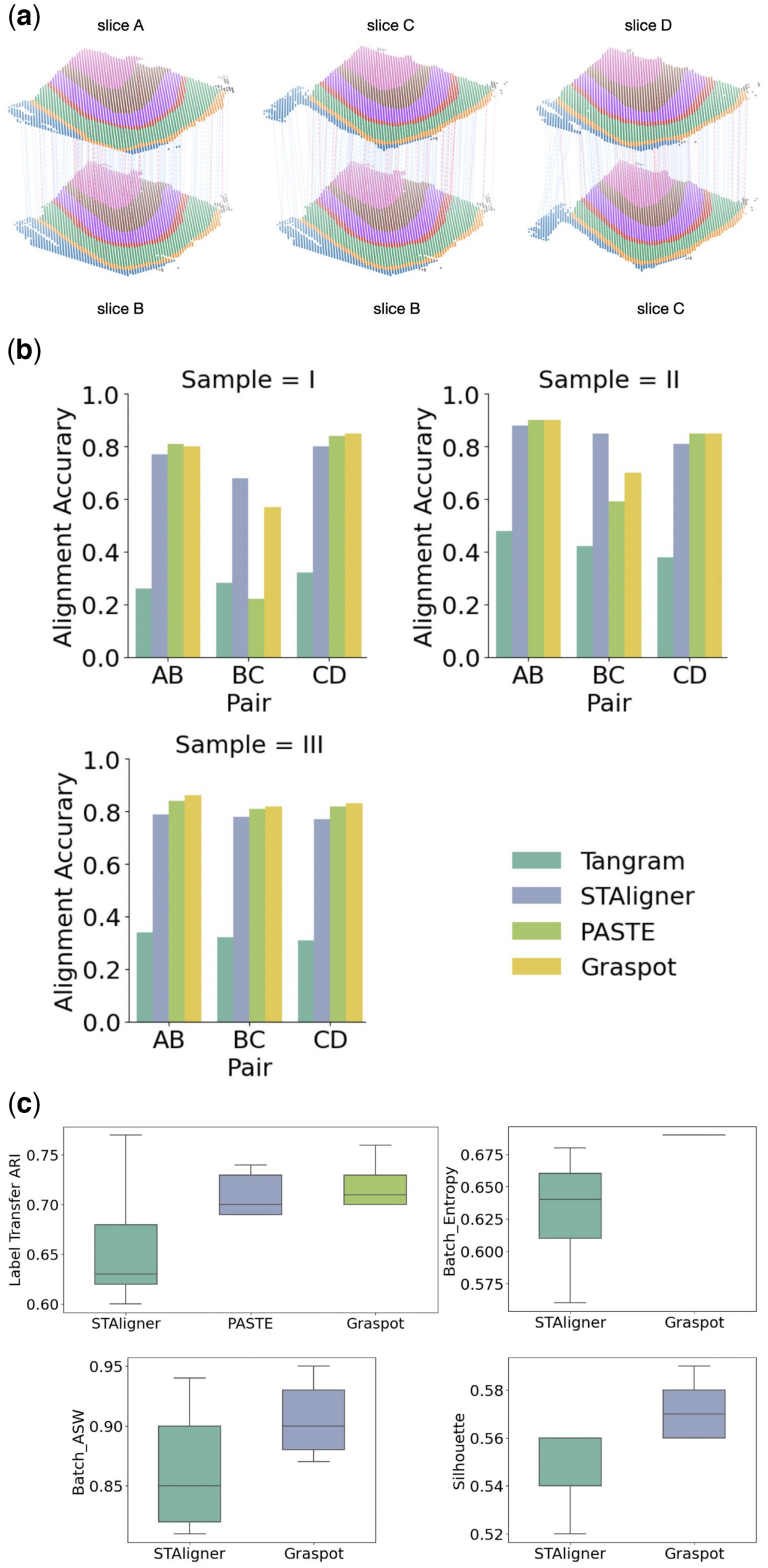
(a) Spot matching results in Sample III using Graspot. Blue lines indicate correct matches, whereas red lines denote incorrect matches. (b) Accuracy of pairwise alignment of consecutive DLPFC slices (labeled AB, BC, and CD) for Tangram, STAligner, PASTE, and Graspot, which is computed based on published annotations of spots. (c) Label Transfer ARI, Batch entropy, Batch ASW, and Silhouette scores of pairwise slices alignment.

We applied Graspot to compute a pairwise slice alignment of each pair of consecutive slices from the same sample. The visualization of parallel spot matching results in Sample III using Graspot is shown in Fig. 2a. We compared the pairwise alignments obtained by Graspot with PASTE (Zeira *et al.* 2022), STAligner (Dong and Zhang 2022), Tangram (Biancalani *et al.* 2021), and the results are shown in the Fig. 2b and c.

Graspot achieved the highest alignment accuracy in 6 of 9 pairwise alignment which shows the efficiency of our method in the alignment tasks in ST of human DLPFC data. As Fig. 2b shows, Graspot obtained a higher accuracy mostly above 0.8 in the close pairs (AB and CD) but lower accuracy in the far pairs (BC). Tangram algorithm achieved lowest accuracy from 0.26 to 0.48 on all slice pairs. STAligner algorithm achieved lower accuracy from 0.77 to 0.88 on all slice pairs, outperforming Graspot only on one middle BC slice pair in Sample I and II. Graspot achieved higher Label Transfer ARI scores with less variation than other methods in nine pairwise alignment tasks. The mean Label Transfer ARI score using Graspot is 0.67, surpassing other compared methods. The results of Batch entropy, Batch ASW, and Silhouette score are shown in Fig. 2c. Graspot is more competitive than STAligner both on removing batch effects and conserving biological structure variations in latent embeddings. Since STAligner cannot provide the probabilistic alignment across slices, we performed a matching strategy in its embedding space for the following evaluation. Due to the lack of a common integrated space in PASTE and Tangram, we only compared our method with STAligner.

These results demonstrate that Graspot, which combines transcriptional and spatial information to align ST data, frequently outperforms algorithms that use only transcriptional information. Additionally, our method, which iteratively learns the embedding and mapping using UOT, exhibits better performance compared to the fused Gromov–Wasserstein optimal transport strategy employed in PASTE and spot triplet learning strategy in STAligner. Figure 2 shows the superiority of Graspot in evaluation of pairwise alignment of consecutive DLPFC slices (labeled AB, BC, and CD) over Tangram, STAligner, and PASTE. It is evident that Graspot improves alignment performance between adjacent slices. For instance, this enhancement is noticeable for all pairs in Sample III and for the BC pairs in both Sample I and Sample II.

### 4.2 Graspot outperforms the state-of-the-art methods on partial alignment with highest accuracies

**Figure 3.**
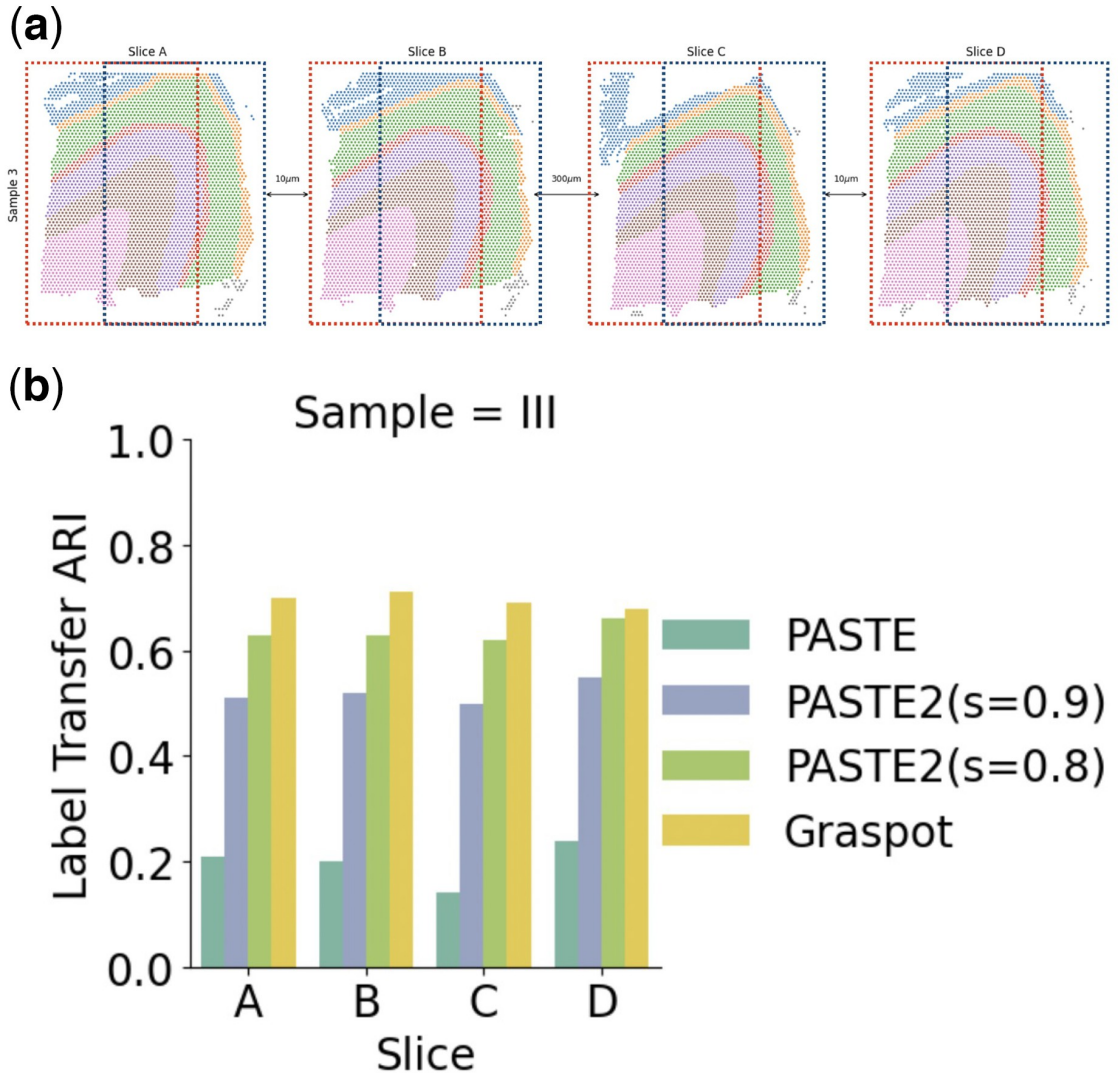
(a) Two vertical subslices, one on the left and the other on the right, exhibit a 70% overlap of spots across four slices from Sample III. (b) Label transfer ARI scores of pairwise alignment of subslices in (a) for PASTE, PASTE2 (*s* = 0.8), PASTE2 (*s* = 0.9), and Graspot.

To evaluate Graspot’s ability to handle unbalanced datasets using UOT module, we tested Graspot on vertical partial subslices of human DLPFC tissues. The slices that we selected are derived from four DLPFC slices in Sample III. For each slice, we generated two partially overlapping subslices in the following manner: selecting two vertical lines on a slice to generate two subslices which encompass 70% of the total spots, as illustrated in Fig. 3a.

Following the strategy of matching to the closest in De Plaen *et al.* (2023), we set $\rho_{1}\to \infty$ and $\rho_{2}=0.01$ for partial alignment. We applied our method to find alignments within the two generated subslices for each of the slices A, B, C, and D in Sample III, separately. We follow PASTE2 (Liu *et al.* 2023) to use the Label Transfer ARI to measure the accuracy of partial alignment: Ground Truth clustering in slice with fewer spots and reordered Ground Truth clustering induced from spot correspondence $\pi (i),i=1,\ldots ,n_{x}$ in another slice. We compared our results with the other two methods (Fig. 3b). Consequently, Graspot demonstrated superior performance with results ranging between 0.68 and 0.71. In contrast, PASTE exhibited lower alignment accuracy ranging from 0.14 to 0.24, attributable to its inability to handle partially overlapping slices. PASTE2 is designed for partial alignment, allowing only a fraction *s* of the probability mass to be transported. Here we set *s* in PASTE2 equal to 0.8. PASTE2 achieved a lower alignment result, ranging from 0.62 to 0.66 compared to Graspot. PASTE2 yielded inferior outcomes with *s *=* *0.9, varying from 0.50 to 0.55. This suggests that PASTE2, when set to a larger *s* value, intends to align the entirety of spots between slices.

### 4.3 Graspot efficiently integrates multiple ST slices via pretraining

The trained graph attention neural network is considered as a reference model which endow Graspot with the capability to iteratively integrate multiple slices into an aligned embedding within a common low-dimensional space. To verify the dominance of handing multiple ST datas, we applied Graspot in four slices of human DLPFC Sample III as follows.

**Figure 4.**
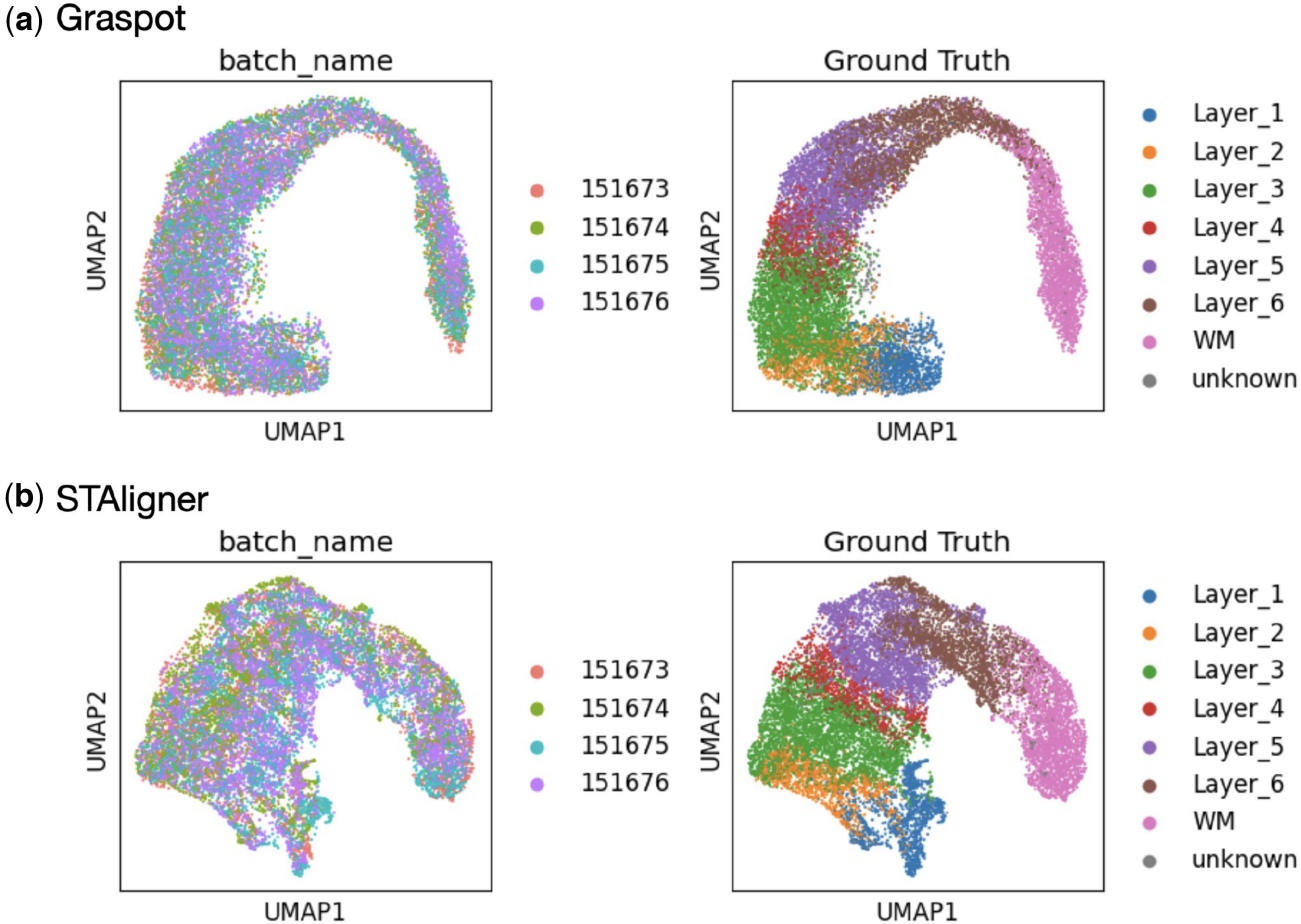
(a) Left: Embedding of four slices in Sample III using Graspot. Right: Clustering results of Graspot. (b) Left: Embedding of four slices in Sample III using STAligner. Right: Clustering results of STAligner.

We trained the neural network thoroughly within our model to align the pairwise slices (Slice B and Slice C) and then input the new slices from the same sample (Slice A and Slice D). As shown in Fig. 4a, the newly inputted slices, including Slice A and Slice D, align well with the existing slice embeddings in the common low-dimensional space. Additionally, the embeddings display clear patterns of cluster results that are in accordance with manually annotated layers. We also integrated the four slices in a common space using STAligner for comparison (Fig. 4b). The embeddings produced by Graspot demonstrate superior alignment and distinct clustering when compared to STAligner, particularly in cases involving the integration of multiple ST slices.

**Figure 5.**
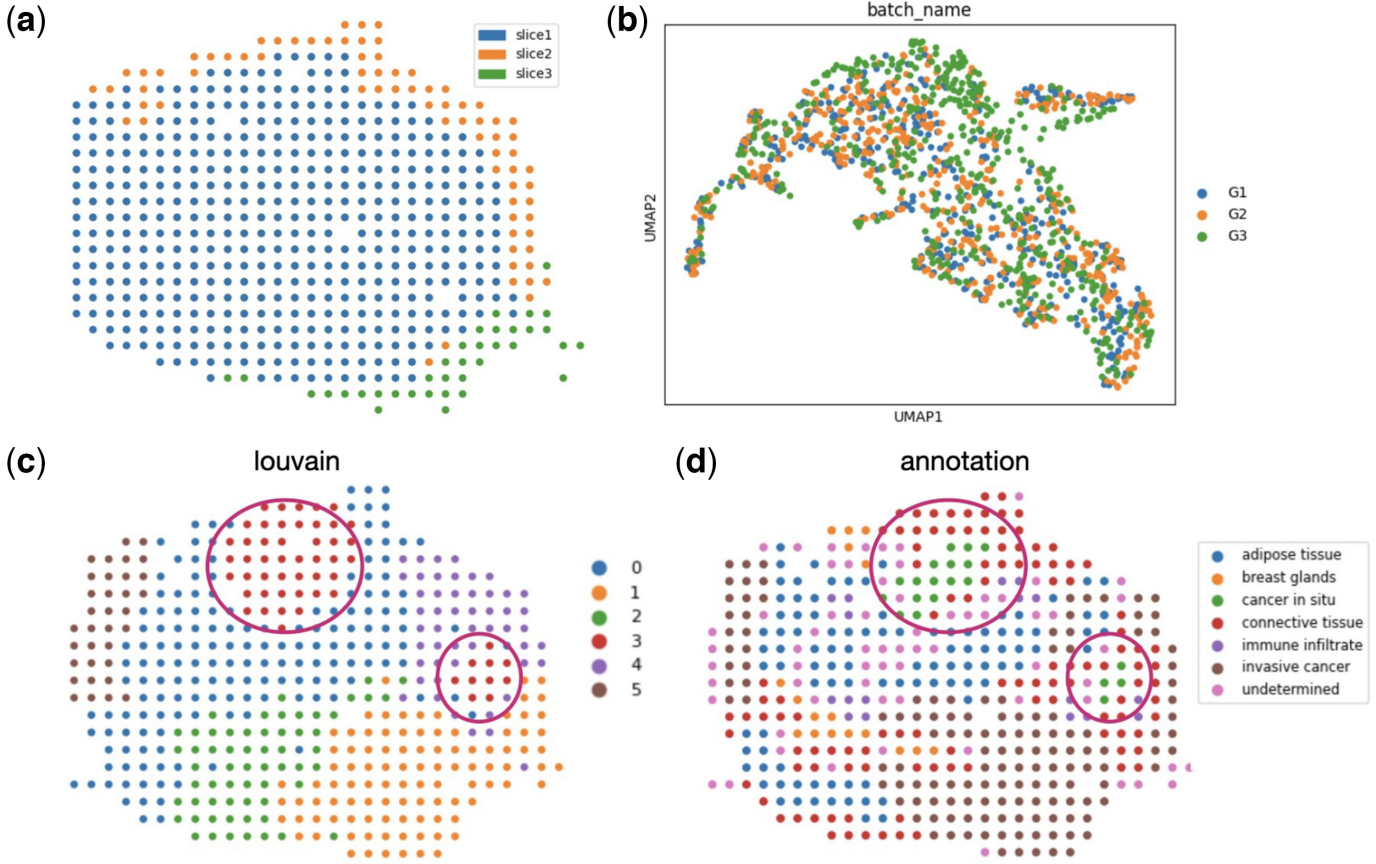
Graspot integrated multiple human breast cancer spatial transcriptomics data. (a) Three spatial transcriptomics slices from HER2 breast cancer patient G with sections of adjacent slices overlapping along the *z*-axis. (b) Integrated embedding of three ST slices in common space. (c) Clusters in template slice G2. (d) Pathological annotations of G2 slice.

We also applied Graspot to analyze three ST slices from HER2 breast cancer of patient G (Andersson *et al.* 2021). The experiment demonstrated that Graspot is capable of effectively performing tasks related to alignment, integration, and identification of distinct spatial structures in biological data. The three ST slices from HER2 breast cancer of patient G are adjacent and part of slices are partial overlap, as depicted in Fig. 5a. Following the alignment of the pairwise slices G1 and G2 using Graspot, the subsequent slice G3 aligns effectively with the existing latent embedding in the shared low-dimensional space. This process results in an integrated embedding, as depicted in Fig. 5b. Afterwards, we employed the Louvain clustering algorithm on integrated spots within the common low-dimensional space. The clustering outcomes for the template slice G2 are displayed in Fig. 5c. Graspot successfully identifies two regions of spots, aligning with the *in situ* cancer regions identified by pathological annotations, as illustrated in Fig. 5d. These results affirm that the integration of multiple ST slices using Graspot effectively preserves and unveils intricate structures within the tumor microenvironment.

### 4.4 Graspot efficiently guides coordinate alignment of ST slices

Without the prior information as anchor pairs, Graspot is able to automatically registrate ST slices in coordinate framework with the guidance of probabilistic alignment *T*. We applied Graspot to analyze one pair of 10× Genomics Visium ST datas from sagittal mouse brain with complex structures.

**Figure 6.**
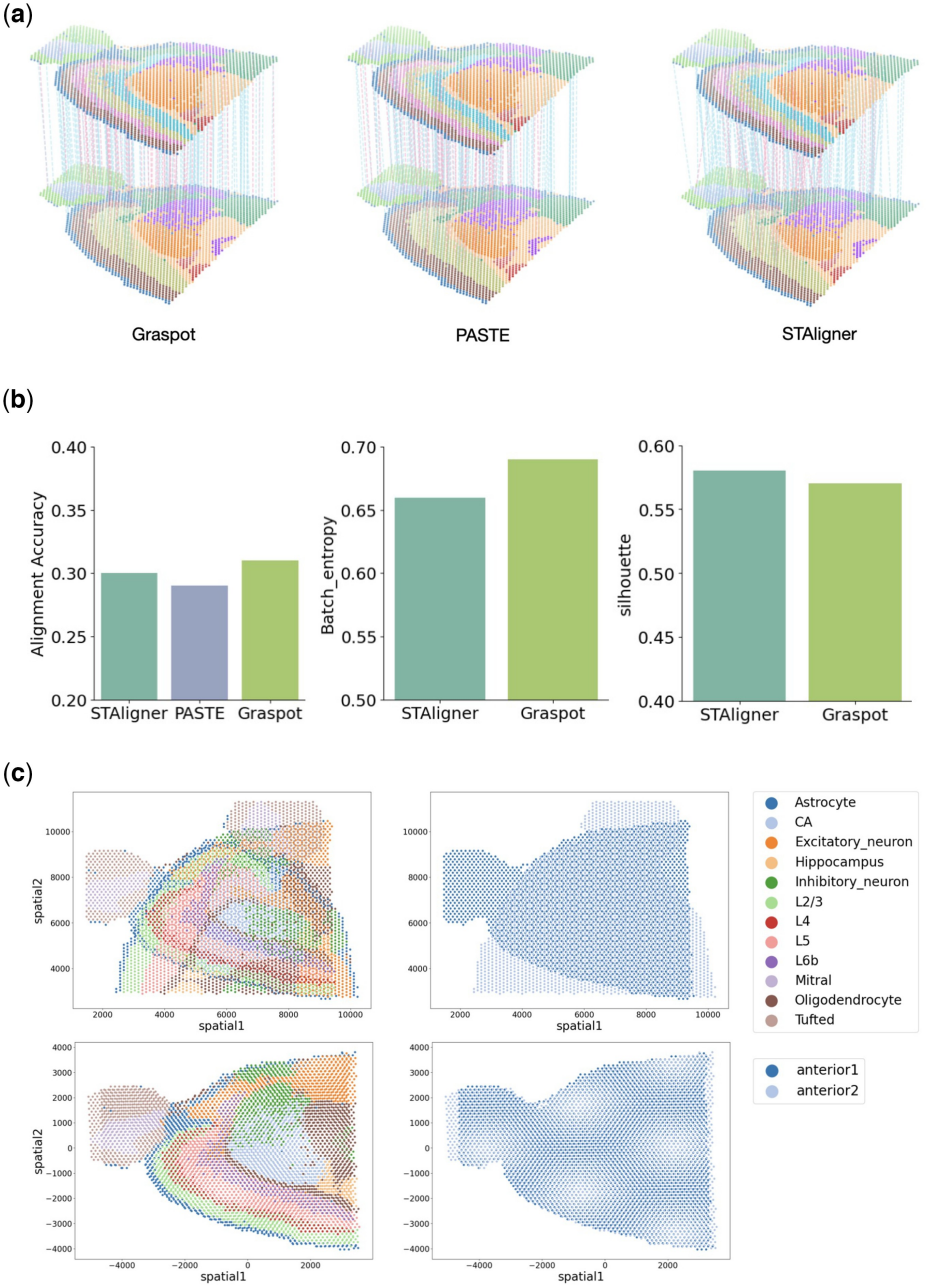
(a) Spot matching results in Sample III using Graspot, PASTE, and STAligner. Blue lines indicate correct matches, whereas red lines denote incorrect matches. (b) Label Transfer ARI, Batch entropy, and Silhouette scores of pairwise slices alignment. (c) Alignment of slices in coordinate with rotation.

Figure 6a depicts the 3D landscape of spot matching results between two replicates of sagittal mouse brain data using Graspot, PASTE, and STAligner, respectively. We benchmarked the performances of methods using the ground truth of annotations which contain common hierarchical layers including “L5,” “L2/3,” “Astrocyte,” and “CA.” We conducted alignment accuracy evaluation in probabilistic spots mapping *T* compared with PASTE and STAligner. Graspot achieved a highest score of 0.31, while STAligner achieved the second highest score of 0.30, and PASTE achieved a lowest score of 0.29 (left panel of Fig. 6b). To assess and demonstrate the integration performance within the common space, we used metrics such as Batch entropy and silhouette score for comparing two methods, namely STAligner and Graspot. Given that PASTE does not offer a common embedding space, it was excluded from these subsequent comparisons. We found that Graspot obtain a higher Batch entropy score than that of STAligner (middle panel of Fig. 6b), while its silhouette score is slightly lower than STAligner (right panel of Fig. 6b).

Given probabilistic spots mapping *T*, Graspot solved weighted ICP algorithm to compute the optimal rotation and translation solution. As shown in Fig. 6c, slice “anterior 2” can be transformed through a 90-degree rotation stitching to slice “anterior 1” which indicates the capability of Graspot in coordinate alignment as one part of downstream analysis.

### 4.5 Graspot partially aligns spatio-temporal ST data of human heart development

**Figure 7.**
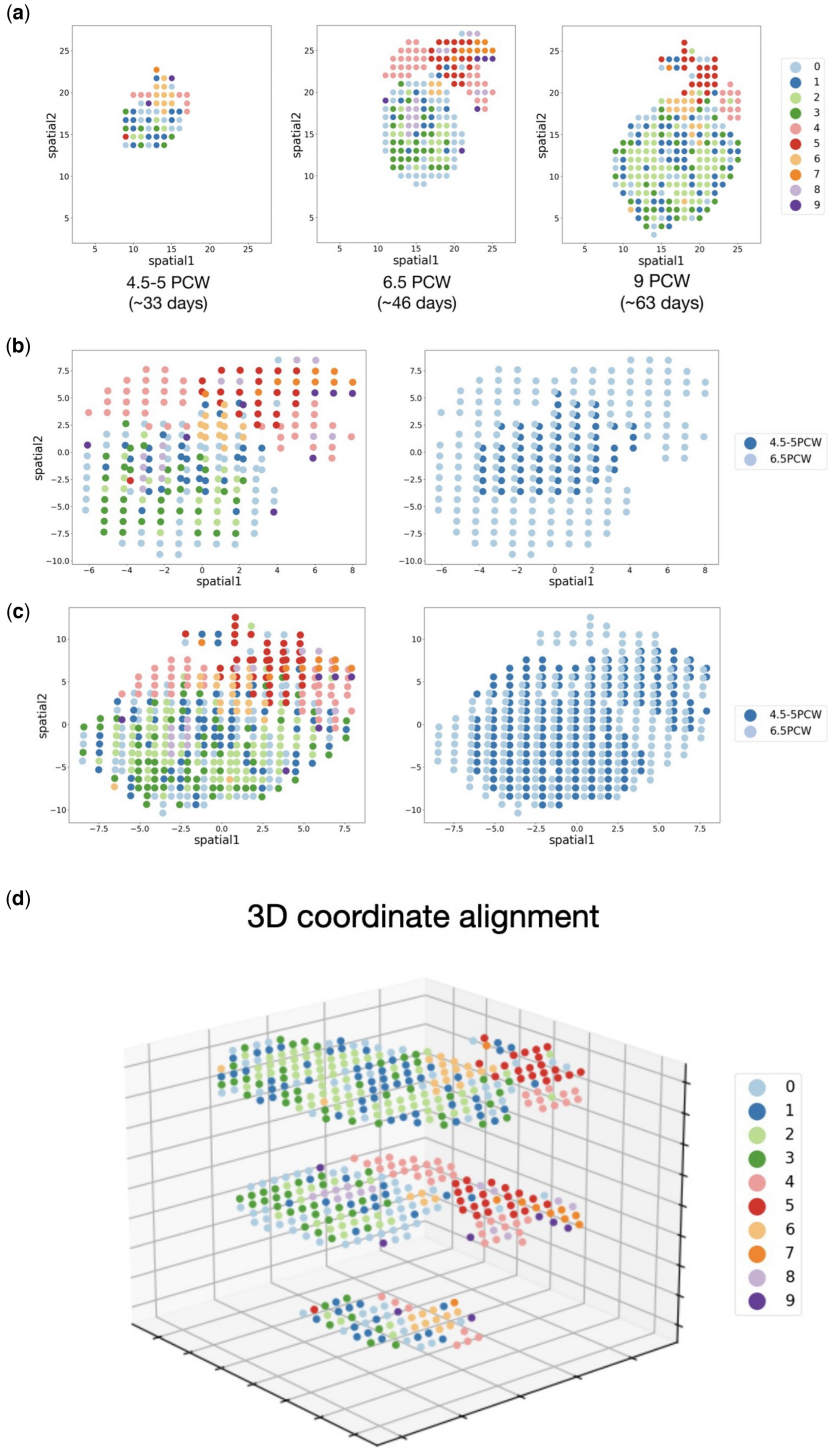
(a) Distribution of spot types in ST slices sampled from three different developmental stage. (b) Coordinate alignment result of ST slices from 4.5 to 5 PCW and 6.5 PCW. (c) Coordinate alignment result of ST slices from 6.5 PCW to 9 PCW. (d) The alignment of three ST slices (4.5–5 PCW, 6.5 PCW, 9 PCW) in a 3D coordinate framework.

Graspot can provide more intuitive insights of spot relationships such as cell growth and differentiation. We applied Graspot to spatio-temporal ST data of human heart development. This dataset contains three slices collected from human heart in early embryos, ranging from 4.5 to 5 post-conception weeks (PCW), 6.5–9 PCW (Asp *et al.* 2019) in Fig. 7a. It is clear that the number of cell types within the ST sections increases as embryonic development progresses (Fig. 7a). For example, the main myocardial region (e.g. cell types 0, 1, 2, 3, 4) grew dramatically between 4.5–5 PCW and 9 PCW, reflecting the heart’s growth processes.

We used the probabilistic alignment matrix **T** output from Graspot to align the coordinates of ST slices at the first two time points and the last two time points, respectively, using the weighted ICP algorithm (Fig. 7b and c). The results show that the aligned overlapping regions of different slices share common cell types, whereas unaligned regions retain slice-specific cell types. This result indicates that Graspot can preserve the common structure of slices during alignment, and provides a basis for further downstream analysis of cell growth and differentiation mechanisms.

We also aligned the three ST slices of human heart development into a 3D coordinate framework. For multiple ST slices alignment, Graspot selects one slice as the reference template, and aligns the other slices with respect to the reference. Here we set the slice of 6.5 PCW as the reference, and aligned the slices of 4.5–5 PCW and 9 PCW to the reference slice. The resulting alignment of ST slices in a 3D coordinate framework is shown in Fig. 7d. It is also clear that the invariant cell types among the three slices (e.g. cell types 2 and 6) are well aligned.

## 5 Discussion

In this study, we introduce Graspot, a GAT designed for the integration of ST data using optimal transport. It generates probabilistic alignments for downstream analysis and aligns the intrinsic structures of multiple slices within a common low-dimensional space. Additionally, Graspot adeptly handles unbalanced alignment by allowing for partial overlap between slices. Our findings demonstrate Graspot’s capability to effectively perform tasks related to alignment, integration, and identification of unique spatial structures. Specifically, Graspot achieves alignments with higher accuracy compared to existing methods on ST data.

It is worth noting that, in the global alignment task of DLPFC dataset, Graspot achieved the second highest alignment accuracy scores in BC pairs of samples I and II (Fig. 2b), surpassed by STAligner. We took a close look at the alignment of BC pair in sample II (Fig. S1 in Supplementary Note S4). We found that Layer4 and Layer5 are invariant across slice B and slice C, whereas Layer3 are expanded from slice B to slice C, Layer6 and WM are contracted from slice B to slice C. The mismatched spots between BC pair (red lines in Fig. S1 in Supplementary Note S4) by Graspot mainly occurs between Layer3 in slice B and WM in slice C. The cross-layer mismatches may be due to the discontinues changes in slice B and slice C. To solve the problem of cross-layer mismatches, we plan to further improve Graspot by incorporating feature information (e.g. fused OT) to penalize the matching across different cell types.

Graspot is a computationally efficient algorithm (Fig. S2 in Supplementary Note S5). However, it requires memory consumption in the storage of optimal transport plan matrices **T**. Therefore, it may not perform well when sample size is in large-scale ST data. Designed for scalability, Graspot can efficiently handle large-scale ST data by employing subgraph sampling and a mini-batch scheme. We plan to explore these topics in our future work.

## Supplementary Material

btae394_Supplementary_Data

## Data Availability

Graspot software is available at https://github.com/zhan009/Graspot. The datasets in this study are all from publicly available datasets. DLPFC dataset is available online at http://research.libd.org/spatialLIBD/. The sagittal mouse brain ST dataset is available online at https://zenodo.org/record/6925603#.YuM5WXZBwuU. HER2 breast cancer ST dataset is available online at https://doi.org/10.5281/zenodo.6334774. And spatio-temporal ST dataset of human heart development is available online at https://www.spatialresearch.org.

## References

[btae394-B1] Andersson A , Larsson L, Stenbeck L et al Spatial deconvolution of HER2-positive breast cancer delineates tumor-associated cell type interactions. Nat Commun 2021;12:6012. 34650042 10.1038/s41467-021-26271-2 PMC8516894

[btae394-B2] Asp M , Giacomello S, Larsson L et al A spatiotemporal organ-wide gene expression and cell atlas of the developing human heart. Cell 2019;179:1647–60.e19. 31835037 10.1016/j.cell.2019.11.025

[btae394-B3] Baccin C , Al-Sabah J, Velten L et al Combined single-cell and spatial transcriptomics reveal the molecular, cellular and spatial bone marrow niche organization. Nat Cell Biol 2020;22:38–48. 31871321 10.1038/s41556-019-0439-6 PMC7610809

[btae394-B4] Benamou J-D. Numerical resolution of an “unbalanced” mass transport problem. Esaim: M2AN 2003;37:851–68.

[btae394-B5] Biancalani T , Scalia G, Buffoni L et al Deep learning and alignment of spatially resolved single-cell transcriptomes with tangram. Nat Methods 2021;18:1352–62. 34711971 10.1038/s41592-021-01264-7 PMC8566243

[btae394-B6] Cao K , Gong Q, Hong Y et al A unified computational framework for single-cell data integration with optimal transport. Nat Commun 2022a;13:7419. 36456571 10.1038/s41467-022-35094-8 PMC9715710

[btae394-B7] Cao K , Hong Y, Wan L. Manifold alignment for heterogeneous single-cell multi-omics data integration using pamona. Bioinformatics 2022b;38:211–9. 10.1093/bioinformatics/btab594 PMC8696097 34398192

[btae394-B8] Cuturi M. Sinkhorn distances: lightspeed computation of optimal transport. Adv Neural Inf Process Syst 2013;26:2292–300.

[btae394-B9] De Plaen H , De Plaen P-F, Suykens JA et al Unbalanced optimal transport: a unified framework for object detection. In: *2023 IEEE/CVF Conference on Computer Vision and Pattern Recognition (CVPR)*, Vancouver, BC, Canada, 2023; 3198–207.

[btae394-B10] Dong K , Zhang S. Deciphering spatial domains from spatially resolved transcriptomics with an adaptive graph attention auto-encoder. Nat Commun 2022;13:1739. 35365632 10.1038/s41467-022-29439-6 PMC8976049

[btae394-B11] Janati H , Muzellec B, Peyré G et al Entropic optimal transport between unbalanced Gaussian measures has a closed form. Adv Neural Inf Process Syst 2020;33:10468–79.

[btae394-B12] Ji AL , Rubin AJ, Thrane K et al Multimodal analysis of composition and spatial architecture in human squamous cell carcinoma. Cell 2020;182:497–514.e22. 32579974 10.1016/j.cell.2020.05.039 PMC7391009

[btae394-B13] Jones A , Townes FW, Li D et al Alignment of spatial genomics data using deep Gaussian processes. Nat Methods 2023;20:1379–87. 37592182 10.1038/s41592-023-01972-2 PMC10482692

[btae394-B14] Liu X , Zeira R, Raphael BJ. Partial alignment of multislice spatially resolved transcriptomics data. Genome Res 2023;33:1124–32. 37553263 10.1101/gr.277670.123 PMC10538490

[btae394-B15] Maynard KR , Collado-Torres L, Weber LM et al Transcriptome-scale spatial gene expression in the human dorsolateral prefrontal cortex. Nat Neurosci 2021;24:425–36. 33558695 10.1038/s41593-020-00787-0 PMC8095368

[btae394-B16] Peyré G , Cuturi M. Computational optimal transport: with applications to data science. FNT Mach Learn 2019;11:355–607.

[btae394-B17] Ståhl PL , Salmén F, Vickovic S et al Visualization and analysis of gene expression in tissue sections by spatial transcriptomics. Science 2016;353:78–82. 27365449 10.1126/science.aaf2403

[btae394-B18] Xie Y , Wang X, Wang R et al A fast proximal point method for computing exact wasserstein distance. In: *Proceedings of the 36th Conference on Uncertainty in Artificial Intelligence (UAI)*, Virtual, PMLR, 2020; 433–53.

[btae394-B19] Zeira R , Land M, Strzalkowski A et al Alignment and integration of spatial transcriptomics data. Nat Methods 2022;19:567–75. 35577957 10.1038/s41592-022-01459-6 PMC9334025

[btae394-B20] Zhou X , Dong K, Zhang S. Integrating spatial transcriptomics data across different conditions, technologies and developmental stages. Nat Comput Sci 2023;3:894–906. 38177758 10.1038/s43588-023-00528-w

