# Supplementary material for: Graspot: a graph attention network for spatial transcriptomics data integration with optimal transport
Source: Bioinformatics. 2024 Sep 4;40(Suppl 2):ii137–45. doi: 10.1093/bioinformatics/btae394 (PMC11520409; doi:10.1093/bioinformatics/btae394)
Supplement: btae394_Supplementary_Data [file btae394_supplementary_data.pdf]

SUPPLEMENTARY INFORMATION

Graspot: A graph attention network for spatial transcriptomics data integration with optimal transport

Zizhan Gao, Kai Cao*, Lin Wan*

Supplementary Notes S1: Graspot algorithm

Algorithm 1 Graspot: a graph attention network for spatial transcriptomics data integration with optimal transport.

Input: Two ST slices $\mathcal{D}_1 = \{(\mathbf{x}_i, \mathbf{s}_i), i = 1, 2, ..., n_x\}$ and $\mathcal{D}_2 = \{(\mathbf{y}_j, \mathbf{w}_j), j = 1, 2, ..., n_y\}$.
Output: Aligned latent vectors $\mathbf{z}_x$ and $\mathbf{z}_y$, an optimal transport plan $\mathbf{T}$.

Build a spatial neighbor network and compute a adjacency matrix $\mathbf{A}$ based on spot locations in two slices respectively.
Initialize GAT encoder ψ and decoder ϕ.
Compute transport cost $\mathbf{C}$ via spatial information and obtain a initial optimal OT plan $\mathbf{T}^{(0)}$.
for $m \leftarrow 1$ **to** M **do**
  a. Input both $(\mathbf{X}^c, \mathbf{S})$ and $(\mathbf{Y}^c, \mathbf{W})$ through the shared graph attention encoder ψ to obtain $\mathbf{z}_x$ and $\mathbf{z}_y$.
  b. reconstruct $\hat{\mathbf{X}}^c$ and $\hat{\mathbf{Y}}^c$ by decoder ϕ.
  c. compute transport cost $\mathbf{C}_{ij}$ via Eq. (6) in latent space and obtain optimal transport UOT alignment plan $\mathbf{T}_B^*$ via Eq. (7).
  d. Fix $\mathbf{T}_B^*$ and update ϕ and ψ through back propagation via minimizing L_{UOT} and L_{Recon}.
  e. Update $\mathbf{T}$ with rows and columns as $\mathbf{T}_B^*$.
end
obtain an optimal transport plan $\mathbf{T}$ and aligned latent vectors $\mathbf{z}_x$ and $\mathbf{z}_y$.

*Corresponding author

Supplementary Notes S2: Details of evaluation metrics

Alignment accuracy

We assume that spot i is in slice A and spot j is in slice B, and Slice A has n_x spots and slice B has n_y spots. The Alignment accuracy evaluation metric is defined as follows:

$$\mathrm{Alignment\_accuracy} = \sum_{\mathrm{i,j}} \mathbf{M}_{\mathrm{ij}} \mathbf{1}(\mathrm{c(i) = c(j)}). \tag{1}$$

When $n_x < n_y$, we compute $\mathbf{M}_{ij}$ by row as follows:

$$\mathbf{M}_{\mathrm{ij}} = \begin{cases} 1 & \forall \mathrm{j}, \mathbf{T}_{\mathrm{ij}} > \mathbf{T}_{\mathrm{i}*} \\ 0 & \mathrm{otherwise} \end{cases}, \tag{2}$$

where $\mathbf{T}_{i*}$ is the i-th row in mapping $\mathbf{T}$ and $c(i)/c(j)$ indicates the annotated or clustered regions which spot i/spot j belongs to.

Label Transfer ARI

Given $\mathbf{M}_{ij}$ which indicates the maximum probability matching, we compute the one-to-one spot correspondence $\pi : n_x \to n_y$. The Label Transfer ARI metric is defined as ARI score of cell-type clusters in sliceA and reorder cell-type clusters in sliceB derived from spot correspondence $\pi(i), i = 1, ..., n_x$. The Adjusted Rand Index (ARI) is utilized to assess the similarity between two different cell-type clusters. The ARI value ranges from -1 to 1, with 0 for random labeling and 1 for perfect matching.

Batch Entropy

Batch entropy score is derived from uniPort [1] which evaluates the sum of regional mixing entropies at the location of randomly chosen spots from different slices. It can be calculated as

$$\mathrm{E} = \sum_{\mathrm{i=1}}^{\mathrm{n}} \mathrm{p}_{\mathrm{i}}' \log(\mathrm{p}_{\mathrm{i}}'), \tag{3}$$

where

$$\mathrm{p}_{\mathrm{i}}' = \frac{\frac{\mathrm{p}_{\mathrm{i}}}{\mathrm{P}_{\mathrm{i}}}}{\sum_{\mathrm{i}=1}^{\mathrm{n}} \frac{\mathrm{p}_{\mathrm{i}}}{\mathrm{P}_{\mathrm{i}}}}, \tag{4}$$

p_i is the proportion of spot numbers in each batch to the total spot numbers, and P_i is the proportion of spots from batch i in a given region. A high Batch entropy score indicates spots from various slices mixing well.

Batch ASW

Batch Average Silhouette Width(Batch ASW) refers to modified silhouette width to measure batch mixing. The Alignment accuracy evaluation metric is defined as follows:

$$\mathrm{Batch\_ASW} = \frac{1}{|\mathrm{M}|} \sum_{\mathrm{j} \in \mathrm{M}} \frac{1}{|\mathrm{C}_{\mathrm{j}}|} \sum_{\mathrm{i} \in \mathrm{C}_{\mathrm{j}}} 1 - |\mathrm{s}_{\mathrm{batch}}^{(\mathrm{i})}|, \tag{5}$$

where M is the set of unique spot labels and $|M|$ denotes the number of spot labels, C_j is the set of spots with the spot label j and $|C_j|$ denotes the number of spots in that set. $|s_{batch}^{(i)}|$ is the silhouette width on batch labels for the i-th spot. Higher Batch ASW indicates ideal mixing.

Silhouette

Silhouette (Cell-type ASW) is used to determine the separation of spot clusters. It is defined as follows:

$$\mathrm{Silhouette} = \frac{1}{2}(\frac{1}{\mathrm{N}} \sum_{\mathrm{i}=1}^{\mathrm{N}} \mathrm{s}_{\mathrm{spot}}^{(\mathrm{i})} + 1), \tag{6}$$

where N presents the total number of spots and $s_{spot}^{(i)}$ is the silhouette width on spot cluster labels for the i-th spot. Higher score of Silhouette represents better biology conservation of the slice integration.

Supplementary Notes S3: 3D reconstruction method

Based on alignment results, we try to find a rotation and translation solution of spatial coordinates in one slice that minimizes the distances to another slice's spatial coordinates. We follow and extend the ICP algorithm in the area of point cloud registration to include

the alignment mapping $\mathbf{T}$ as in PASTE[2].

Given alignment mapping $\mathbf{T}_{ij} \in \Gamma(\mathbf{a}, \mathbf{b})$ and a static slice's spatial coordinates $\mathbf{S} \in \mathbb{R}^{2\times n_x}$ and a transformed slice's spatial coordinates $\mathbf{W} \in \mathbb{R}^{2\times n_y}$, the problem can be solved by finding optimal transformation including rotation matrix $\mathbf{R}$ and translation vector $\mathbf{t}$ as follows:

$$\mathbf{R}^*, \mathbf{t}^* = \mathrm{argmin}_{\mathbf{R},\mathbf{t}} \sum_{\mathrm{i,j}} \mathbf{T}_{\mathrm{ij}} ||\mathbf{s}_{\mathrm{i}} - \mathbf{R}\mathbf{w}_{\mathrm{j}} - \mathbf{t}||^2. \tag{7}$$

First we calculate the optimal translation to center the spatial coordinates $\mathbf{S}$ and $\mathbf{W}$. By taking the derivative of objective function $\mathrm{F}(\mathbf{R}, \mathbf{t}) = \sum_{\mathrm{i,j}} \mathbf{T}_{\mathrm{ij}} ||\mathbf{s}_{\mathrm{i}} - \mathbf{R}\mathbf{w}_{\mathrm{j}} - \mathbf{t}||^2$ w.r.t. $\mathbf{t}$ to zero:

$$\frac{\partial \mathrm{F}}{\partial \mathbf{t}} = -2\sum_{\mathrm{i}} \mathbf{s}_{\mathrm{i}}\mathrm{a}_{\mathrm{i}} + 2\mathbf{R}\sum_{\mathrm{j}} \mathbf{w}_{\mathrm{j}}\mathrm{b}_{\mathrm{j}} + 2\mathbf{t} = 0, \tag{8}$$

we get optimal translation vector $\mathbf{t}$ as

$$\mathbf{t}^* = \mathbf{S}\mathbf{a} - \mathbf{R}\mathbf{W}\mathbf{b}. \tag{9}$$

New coordinates after translation are defined as $\mathbf{s}_{\mathrm{i}} = \mathbf{s}_{\mathrm{i}} - \mathbf{S}\mathbf{a}$ and $\mathbf{w}_{\mathrm{j}} = \mathbf{w}_{\mathrm{j}} - \mathbf{W}\mathbf{b}$ respecitvely. Next we derive the formulas for optimal rotation matrix. Regardless translation matrix $\mathbf{R}$, objective function $F(\mathbf{R})$ is defined as

$$\begin{aligned}
\mathrm{F}(\mathbf{R}) &= \sum_{\mathrm{i,j}} \mathbf{T}_{\mathrm{ij}} ||\mathbf{s}_{\mathrm{i}} - \mathbf{R}\mathbf{w}_{\mathrm{j}}||^2 \\
&= -2\sum_{\mathrm{i,j}} \mathbf{T}_{\mathrm{ij}} \mathbf{s}_{\mathrm{i}}^{\mathrm{T}} \mathbf{R}\mathbf{w}_{\mathrm{j}} + \beta \\
&= -2\mathrm{trace}(\mathbf{T}^{\mathrm{T}}\mathbf{S}^{\mathrm{T}}\mathbf{R}\mathbf{W}) + \beta,
\end{aligned} \tag{10}$$

where β is an instant which is independent of $\mathbf{R}$. Based on matrix property trace($\mathbf{AB}$) = trace($\mathbf{BA}$), we use SVD to decompose $\mathbf{H}$ into $\mathbf{U}\mathbf{\Sigma}\mathbf{V}^T$ in $F(\mathbf{R})$ and then get

$$\begin{aligned}
\mathrm{F}'(\mathbf{R}) &= -2\mathrm{trace}(\mathbf{T}^{\mathrm{T}}\mathbf{S}^{\mathrm{T}}\mathbf{R}\mathbf{W}) \\
&= -2\mathrm{trace}(\mathbf{R}\mathbf{W}\mathbf{T}^{\mathrm{T}}\mathbf{S}^{\mathrm{T}}) \\
&= -2\mathrm{trace}(\mathbf{R}\mathbf{H}) \\
&= -2\mathrm{trace}(\mathbf{R}\mathbf{U}\mathbf{\Sigma}\mathbf{V}^{\mathrm{T}}) \\
&= -2\mathrm{trace}(\mathbf{\Sigma}\mathbf{V}^{\mathrm{T}}\mathbf{R}\mathbf{U}) \\
&= -2\mathrm{trace}(\mathbf{\Sigma}\mathbf{M}),
\end{aligned} \tag{11}$$

where $\sigma_i \geq 0$ and $m_{ii} \leq 1$. So we get optimal rotation matrix $\mathbf{R}$ as

$$\mathbf{V}^{\mathrm{T}}\mathbf{R}^*\mathbf{U} = \mathbf{I}, \tag{12}$$

$$\mathbf{R}^* = \mathbf{V}\mathbf{U}^{\mathrm{T}}. \tag{13}$$

Supplementary Notes S4: Pairwise alignment of BC pair in Sample II

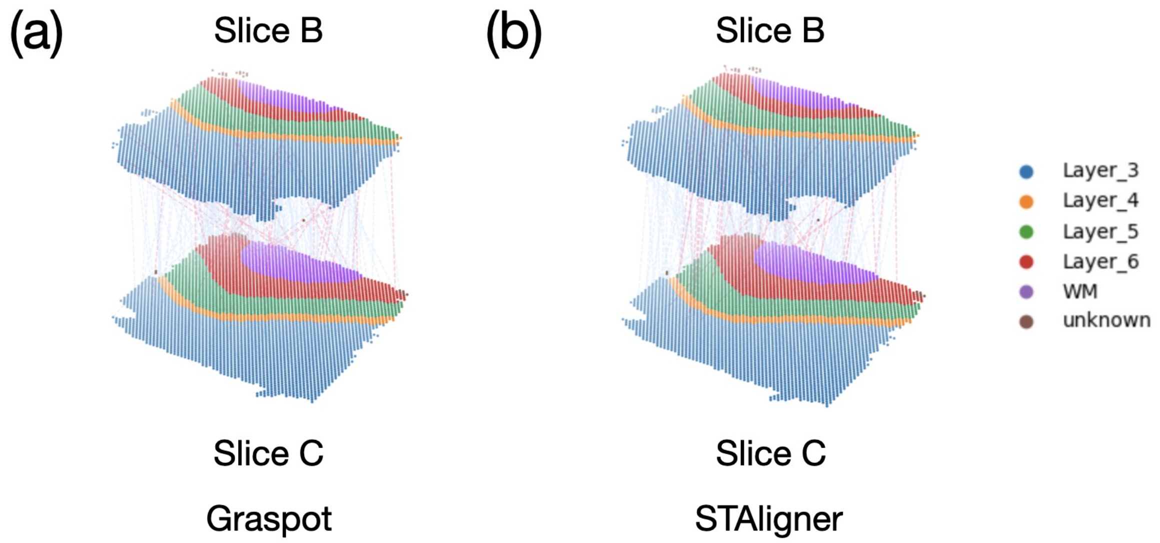

Figure 1: Pairwise alignment results of BC pair in Sample II using Graspot and STAligner respectively.

Supplementary Notes S5: Computational time comparision

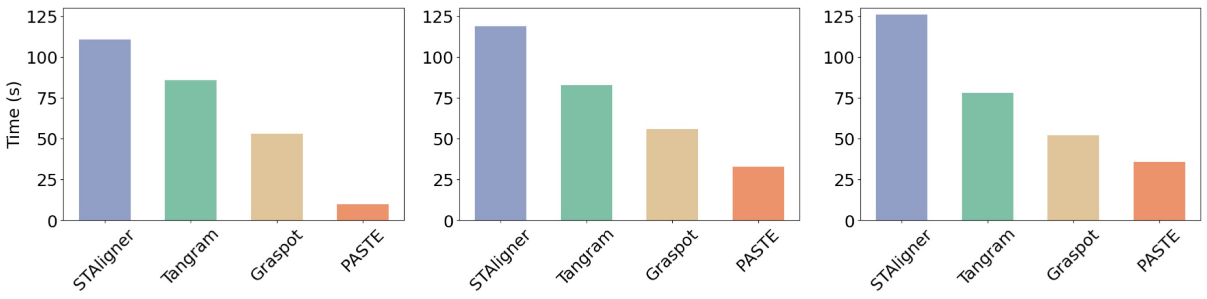

Figure 2: Running time of four different methods on DLPFC ST slices in Sample III.

References

[1] Kai Cao, Qiyu Gong, Yiguang Hong, and Lin Wan. A unified computational framework for single-cell data integration with optimal transport. *Nature Communications*, 13(1):7419, 2022.

[2] Ron Zeira, Max Land, Alexander Strzalkowski, and Benjamin J Raphael. Alignment and integration of spatial transcriptomics data. *Nature Methods*, 19(5):567–575, 2022.
